# Critical views on postpartum care expressed by new mothers

**DOI:** 10.1186/1472-6963-7-178

**Published:** 2007-11-05

**Authors:** Ann Rudman, Ulla Waldenström

**Affiliations:** 1 Department of Woman and Child Health, Karolinska Institutet, Campus Solna, Retzius väg 13, 171 77 Stockholm, Sweden

## Abstract

**Background:**

Women's evaluation of hospital postpartum care has consistently been more negative than their assessment of other types of maternity care. The need to further explore what is wrong with postpartum care, in order to stimulate changes and improvements, has been stressed. The principal aim of this study was to describe women's negative experiences of hospital postpartum care, expressed in their own words. Characteristics of the women who spontaneously gave negative comments about postpartum care were compared with those who did not.

**Methods:**

Data were taken from a population-based prospective longitudinal study of 2783 Swedish-speaking women surveyed at three time points: in early pregnancy, at two months, and at one year postpartum. At the end of the two follow-up questionnaires, women were asked to add any comment they wished. Content analysis of their statements was performed.

**Results:**

Altogether 150 women gave negative comments about postpartum care, and this sample was largely representative of the total population-based cohort. The women gave a diverse and detailed description of their experiences, for instance about lack of opportunity to rest and recover, difficulty in getting individualised information and breastfeeding support, and appropriate symptom management. The different statements were summarised in six categories: organisation and environment, staff attitudes and behaviour, breastfeeding support, information, the role of the father and attention to the mother.

**Conclusion:**

The findings of this study underline the need to further discuss and specify the aims of postpartum care. The challenge of providing high-quality follow-up after childbirth is discussed in the light of a development characterised by a continuous reduction in the length of hospital stay, in combination with increasing public demands for information and individualised care.

## Background

Women's evaluation of postpartum care has consistently been more negative than their assessment of other episodes of maternity care [1-5]. Despite such evaluations, postpartum care is often given low priority in research and practice [6-8]. Considering that patients in general are reluctant to express critical comments about the care they receive, the negative assessments of postpartum care in modern obstetric units are of great concern [9-12]. Important aspects reported to be associated with experiences of postpartum hospital care are the content and provision of information, support and follow-up, communication with caregivers, care facilities, as well as staff attitudes, behaviour, and competence [1,2,13-19].

### Studying negative experiences of health care

A growing interest in taking the user perspective into account when evaluating and planning health care services [20] has resulted in a wide range of literature in the field of patient satisfaction [21]. The aim of this research is to measure quality of care, and to understand patients' experiences [22]. Because these studies have usually presented very high satisfaction scores, their relevance has been questioned on the grounds that they disguise unfavourable experiences and fail to pinpoint less effective areas of care where improvements could be made [11,21]. Service users' uncritical evaluations suggest that they are prepared to accept very poor quality of care before they express dissatisfaction [11,23]. This reluctance to make critical assessments has been explained with reference to patients' perception of gratitude, unwillingness to express critical views (social unacceptability), indifference, loyalty or confidence in the health care system [10,12,21]. Features underlying reports of negative experiences of health care were disempowerment, dehumanisation and devaluation, all of which are experiences that may challenge personal identity and undermine the sense of self [24]. Suggestions have been made to further explore what is wrong with care, in order to form a basis for change [25-27] and improvement of, for example, lay-practitioner relationships [28].

Qualitative approaches have been suggested as a complement to quantitative assessments, in order to increase understanding about the patient perspective on care received [20,29,30]. The present study is a complement to two previously published quantitative studies on women's hospital care after the birth, based on a national sample of Swedish-speaking women [4,5]. Open-ended questions in two postal questionnaires, filled in at two months and one year after the birth, were analysed using content analysis with a focus on critical comments only. This approach was motivated by our interest in finding comments that could form the basis for future improvements of postpartum care.

Women's descriptions of their negative experiences of postpartum hospital care are reported. Characteristics of the women who spontaneously gave negative comments about postpartum care are also compared with those of the women who did not. These comparisons included sociodemographic background, labour outcomes, care organisation and overall assessment of postpartum care.

A growing interest in taking the user perspective into account when evaluating and planning health care services [20] has resulted in a wide range of literature in the field of patient satisfaction [21]. The aim of this research is to measure quality of care, and to understand patients' experiences [22]. Because these studies have usually presented very high satisfaction scores, their relevance has been questioned on the grounds that they disguise unfavourable experiences and fail to pinpoint less effective areas of care where improvements could be made [11,21]. Service users' uncritical evaluations suggest that they are prepared to accept very poor quality of care before they express dissatisfaction [11,23]. This reluctance to make critical assessments has been explained with reference to patients' perception of gratitude, unwillingness to express critical views (social unacceptability), indifference, loyalty or confidence in the health care system [10,12,21]. Features underlying reports of negative experiences of health care were disempowerment, dehumanisation and devaluation, all of which are experiences that may challenge personal identity and undermine the sense of self [24]. Suggestions have been made to further explore what is wrong with care, in order to form a basis for change [25-27] and improvement of, for example, lay-practitioner relationships [28].

Qualitative approaches have been suggested as a complement to quantitative assessments, in order to increase understanding about the patient perspective on care received [20,29,30]. The present study is a complement to two previously published quantitative studies on women's hospital care after the birth, based on a national sample of Swedish-speaking women [4,5]. Open-ended questions in two postal questionnaires, filled in at two months and one year after the birth, were analysed using content analysis with a focus on critical comments only. This approach was motivated by our interest in finding comments that could form the basis for future improvements of postpartum care.

Women's descriptions of their negative experiences of postpartum hospital care are reported. Characteristics of the women who spontaneously gave negative comments about postpartum care are also compared with those of the women who did not. These comparisons included sociodemographic background, labour outcomes, care organisation and overall assessment of postpartum care.

### Aim

The principal aim of this study was to describe negative experiences of postpartum care in women who were given the opportunity to write down any thoughts or comments they wished, at the end of two comprehensive questionnaires about experiences of childbirth and satisfaction with maternity care, one posted at two months and the other at one year after the birth. Also, the women who spontaneously gave negative comments about postpartum care were compared with those who did not, with respect to sociodemographic background, labour outcomes, care organisation, and overall assessment of postpartum care, measured quantitatively by a question with predefined response alternatives.

## Methods

### Participants

The context of this study was the KUB study (Kvinnors upplevelse av barnafödande = Women's experiences of childbirth), a large prospective longitudinal study investigating women's experiences of pregnancy and childbirth from a wide range of perspectives [4,31-34]. Our aim was to invite the participation of all Swedish-speaking women in Sweden, at their first antenatal care clinic visit in early pregnancy, during three one-week periods evenly spread over one year (i.e. May and September 1999 and January 2000). Of 4600 eligible women, 3455 (75%) consented to participate in the study. The women received three questionnaires at three time-points, i.e. in early pregnancy (T1, n = 3061), two months postpartum (T2, n = 2762) and one year postpartum (T3, n = 2563). The background characteristics of the respondents at T1 were compared with those of the total Swedish birth cohort of 1999, including 84,729 women (these data were retrieved from the Swedish Medical Birth Register) [4]. The major difference was that a larger proportion of the KUB cohort was born in Sweden (10% vs. 17% in the total national birth cohort). It should be noted, however, that the respondents to the KUB study were not removed from the Swedish birth cohort of 1999 when pursuing the comparison analyses.

For the purpose of this study, we included only women who gave negative comments about postpartum care when presented with the following two questions at the end of the two questionnaires:

- Two months postpartum: *"If you like you can write down your thoughts and reflections here."*

- One year postpartum: *"If you want to add something, please write your thoughts and opinions below."*

**Figure 1:**
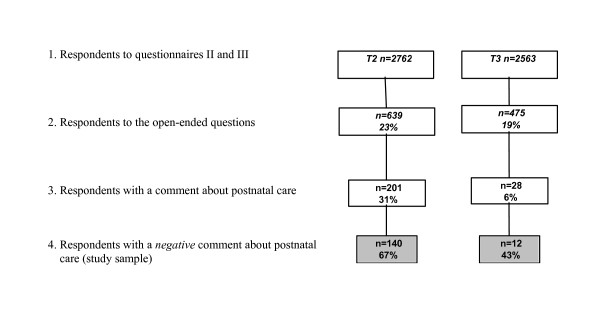
Number of participants in the KUB study in total, and number of respondents to the open-ended questions asked at two months (T2) and one year (T3) after the birth. Note: Total n = 150 (2 women responded at both time points).

The response rate to the first question was 23 percent, and to the second 19 percent, of those who returned the respective questionnaire (Figure 1). In total, 192 women commented on their postpartum care experience, a minority of them with positive statements (n = 41, 21%), and a majority with negative statements (n = 150, 78%). Of the 150 women with a negative comment, who constituted the present study group, 30 (20%) also mentioned positive aspects of postpartum care. Two women responded with a negative comment at both time point 2 and time point 3, and the texts of both their comments were analysed as one. In this way the risk of duplicating the same concern twice and giving greater weight to some areas than to others was avoided.

Ethical approval for the study was obtained from the Regional Research and Ethics Committee at Karolinska Institutet, Sweden (DNR 98-358).

### Procedure of analysis

#### Content analysis

The handwritten comments of all 1114 women (n = 693+475, Figure 1) who responded to the two open-ended questions were scanned into a Word document and read through. Content that did not address postpartum care was then excluded. Comments relating to postpartum care were transferred into a separate file, read through several times, sorted and labelled according to the technique of content analysis [35,36]. Text relating to descriptions of negative and positive aspects of postpartum care was identified. For the purpose of this study the positive meaning units were excluded. Both positive and negative comments were found in 30 women. In accordance with the focus of the study, only the negative meaning units expressed by women with both negative and positive statements were included in the subsequent content analysis. All the negative statements were read through again several times by the first author. Meaning units were labelled with codes, and codes were collapsed into categories. Six categories were generated, mainly based on the visible and descriptive level of the text content (i.e. the manifest content). The coding and category scheme was discussed with the second author throughout the entire process, and modifications and revisions were made when data did not allow simple and clear interpretation. Constantly comparing statements from the comments, and moving back and forth between the text and category scheme, contributed to the refinement and validation of the classification system. The procedure of condensing meaning units was limited, since the original statements were relatively condensed. Due to the inherent complexity of experiences of care, certain comments could be classified to more than one category. However, the overlap of categories was discussed between the authors and kept to a minimum. In order to justify the findings and verify the categories, the descriptions of care were kept close to the original comments, and quotations were used to exemplify the categories.

Statements were defined as negative when they described the absence of expected care, such as lack of follow-up, help, information and support. Statements alluding to how care was given, including poor or unsympathetic manner of the caregiver, were also defined as negative; i.e., both descriptions of what was missing or wrong, and negative experiences of the way it was given, were included.

Statements were defined as positive when women praised the care they were given for fulfilling their needs or expectations. This was, for example, expressed by descriptions of care being helpful, supportive, encouraging or informative. Also, statements that described care as being given in a pleasant, reassuring or supportive manner were defined as positive. The majority of statements defined as positive were of a general nature, including explicit descriptions, such as "care was good" or "care given on the mother's terms".

#### Background data

The rationale for comparing all 150 women who gave a negative statement (subsequently used in the content analysis) with the remaining KUB sample was that they were the focus of interest in this study by having written down their negative experiences. The inclusion of women with both negative and positive statements in the background comparison should be kept in mind when reading Table 1. Questions about sociodemographic background (age, parity, smoking, marital status, country of birth, support partner) were included in the first questionnaire of the KUB study, which was completed in the second trimester (average gestational week 16). Questions about labour outcomes (mode of delivery, newborn transfer to neonatal clinic), care organisation (length of postpartum stay, model of postpartum care, having talked through the birth experience), and satisfaction with postpartum care, were included in the second questionnaire posted two months after the birth. The assessment of postpartum care overall was based on a single item question worded 'What was your comprehensive assessment of postpartum care?' and the five response alternatives ranged from 'very positive' to 'very negative'. The background data of the entire KUB sample are described in detail elsewhere [4,5]. Information about hospital size was collected from the Swedish medical birth register [37].

**Table 1:** Characteristics of the study sample* and the total KUB population**

**Table 1**			**Study sample N = 150%**	**KUB population N= 2633%**	**p**
**Sociodemographic Background**	**Maternal age, years**	Mean	30.05	29.45	ns
		Standard deviation (SD)	SD 4.541	SD 4.676	
		< 25	14	15	ns
		25–35	75	75	
		> 35	11	10	
	**Education**	9-year compulsory school	4	6	ns
		Upper secondary school	51	55	
		College/University	45	39	
	**Parity**	Primipara	48	43	ns
		Multipara	52	57	
	**Smoking**	No	86	90	ns
		Yes	14	10	
	**Marital status**	Married/cohabiting	95	95	ns
		Single/other	5	5	
	**Country of birth**	Sweden	94	91	ns
		Other than Sweden	6	9	
	**Experience of support by partner**	All support and almost all support	93	95	ns
		Little or no support	7	5	

**Labour outcomes**	**Mode of delivery**	Normal vaginal	79	81	ns
		Instrumental vaginal	13	12	
		Elective CS	5	5	
		Emergency CS	3	2	
	**Newborn transfer to NEO**	No	86	89	ns
		Yes	14	11	

**Care organisation**	**Hospital size (number of deliveries/year)**	< 500	3	4	0.02
		500–999	5	13	
		1000–1999	28	28	
		2000–2999	21	20	
		3000–3999	20	20	
		> 4000	23	15	
	**Length of postpartum stay**	< 1 day	12	10	ns
		2 days	22	21	
		3 days	22	25	
		4 days	24	21	
		> 5 days	20	23	
	Model of postpartum care***	Standard pp care	53	51	ns
		Combined ip/pp care	4	8	
		Family pp care	23	23	
		Risk pp care	3	3	
		Hotel pp care	10	10	
		Other	7	5	
	**Talked through the birth experience**	Yes	43	52	0.05
		No	57	48	

**Care evaluation**	**General assessment of postnatal care**	Very positive	14	36	
		Positive	37	40	
		Neither positive or negative	21	18	
		Negative	19	5	
		Very negative	9	1	

ns = non-significant difference.

*Study sample = 150 women who gave a negative comment about postpartum care on the open-ended questions 140 + 10 (figure 1).

**KUB population = the large study (KUB) population with the exclusion of the 150 women in the study sample.

***Models of postpartum care: Standard pp care = standard postpartum care in hospital. Comb ip/pp care = combined delivery and postpartum ward. Family pp care = family-oriented postpartum ward in the hospital where the father can stay overnight. Risk pp care = high-risk postpartum ward. Hotel pp care = postpartum care in a hotel located in close proximity to the delivery ward. Other = other options, for example birth-centre care or discharge directly from the delivery ward.

#### Statistical analysis

SPSS version 15.0 was used for statistical analysis [38]. Descriptive statistics were used to portray the characteristics of participants and their general assessment of care (Table 1). Statistical analyses involved comparison between the study sample and those who participated in the KUB study without a negative comment. On categorical variables, Chi-Square tests and t-tests were carried out to compare groups with regard to proportions and continuous variables respectively. Statistical significance was set at a *p *value of < 0.05.

## Results

The characteristics of the study sample, compared with the remaining participants in the KUB cohort, are presented in Table 1. The study group was largely representative of the KUB cohort, but postpartum care in a large hospital (> 4000 deliveries/year), and not having talked through the birth experience, were more common in women who gave negative comments about postpartum care. Despite having written about a particular negative experience, 51 % rated postpartum care overall as "very positive" (14%) or "positive" (37%). Furthermore, 21% assessed postpartum care as being "neither positive nor negative", 19% as "negative" and 9 % as "very negative".

### Six categories

Six categories of negative statements emerged from the analysis, relating to: organisation and environment, staff attitudes and behaviour, breastfeeding support, information, the role of the father and attention to the mother (Table 2). The category "attention to the mother" was the most mentioned category, including a wide range of experiences. The least stated category was "the role of the father".

**Table 2:** Areas of postpartum hospital care associated with women's negative concerns.

**Areas of postpartum hospital care**
**1. Organisation and environment (n = 77)**
Shortage of staff
Lack of staff continuity
Shortage of beds
Deficient physical environment in general
Inflexible length of stay
Fathers not allowed to stay overnight
Separation when baby is at neonatal clinic
Problems experienced in postpartum care at a hotel
**2. Staff attitudes and behaviour (n = 71)**
Lack of interest, invisible
Insensitive, unfriendly, disrespectful, impersonal
Incompetent
Rushed, stressed
**3. Breastfeeding support (n = 37)**
Insufficient support
Inappropriate support
**4. Information (n = 47)**
Insufficient
Inconsistent
Incorrect
**5. The role of the father (n = 8)**
Lack of attention and support
**6. Attention to the mother (n = 91)**
Insufficient attention to physical health
Insufficient attention to emotional needs

### Organisation and environment

Respondents ascribed many negative experiences to deficient care organisation, e.g. under-funding and policy issues. Several mothers attributed an unwelcoming and irritated atmosphere to *staff shortages *and lack of time.

*As there are so few staff at hospitals these days, the service is getting worse. The midwives really try to make the best of the situation, but they're rushed and haven't got time.... What I mean is that more staff are needed so that you feel secure and "dare" to ask everything you want, without feeling "stupid"*.

*Lack of staff continuity *was a problem when women met too many caregivers, and when they seemed to be unaware of what their colleagues had already said or done.

*I think it was negative that there were so many midwives coming and going when I was there. They all had different views about how to breastfeed etc. I think that if you work together you should be able to give more or less the same answer*.

I think the help they gave at the maternity ward was bad. When about seven nurses (different ones) had been in my room to help me (all these people coming and going made me very stressed)

Women seemed to understand that the prerequisites for providing high quality care were limited because of overcrowded wards due to *shortage of beds*. Sometimes a woman had to sleep in the corridor, and overcrowded rooms and lack of single rooms impacted on privacy, and during summertime, the wards were described as chaotic.

Dissatisfaction was also expressed in relation to *deficient physical environment in general*, such as bad mattresses, shabby rooms and the fact that the room temperature was too high, which made mothers feel uncomfortable.

*There were no curtains around the beds, and there was no special room for breastfeeding. When I had to share a room with another woman, I had the choice of sitting there in front of her and her family when I was hand-milking, or locking myself in the toilet and blocking it for everyone else for half an hour now and then*.

Noise, stressful atmosphere, and people running in and out of doors made it difficult to rest, sleep and recover after the birth.

*After the delivery (where I had a really big incision, which was painful, and ruptures) I ended up in a 2-bed room in the maternity ward. Because they were short of beds there were three of us in that tiny little room. In the first three days I slept perhaps ten hours in all. My stay at the maternity ward was extremely tough and made me depressed. The staffs were pleasant, but that doesn't compensate for the terrible environment and the fact that I couldn't get any sleep! = negative experience*.

Inappropriate routines on the postpartum ward, for example, lack of support during night-time was a problem, especially in combination with the father having been sent home. Absence of routines resulted in a sense of unstructured care.

Shortage of beds was understood as a reason for having to leave hospital too early. *Inflexible length of stay*, not adapted to individual needs, was described as an important issue. The feeling of being "kicked out" or pressured into leaving hospital only a few hours after the delivery caused stress and made women feel that their individual requirements of support were not met.

I think that the time you stay at the maternity ward today is much too short. This is my fourth child and the first time I've been kicked out after 1 1/2 days..... If it's your first baby perhaps you need more support and guidance than it's possible to give in one day, and if like me you're a mother of several children you need rest, and peace and quiet

On the other hand, other respondents said that their request to go home early was not met because their baby had been transferred to the neonatal clinic, or because they had to wait for test results, or because the staff had negative attitudes to early discharge (sometimes it felt as if this was related to young maternal age).

Some mothers were distressed when the *father was not allowed to stay overnight*, or when they were uncertain about whether he could stay or not.

*I felt pretty deserted when my husband had to go home at 4.30 in the morning, and I was pushed into a ward at the maternity department with a little mite who I had hardly even held myself*.

*Being separated from the baby *was stressful and also made it difficult to establish contact with the staff at the postpartum ward. One woman described that it was hard having one's baby in the neonatal clinic and at the same time sharing a room with a mother who roomed-in with her baby.

A model of care associated with many negative comments was *postpartum care at a hotel *(i.e. postpartum care in a patient hotel located in close proximity to the delivery ward. In most cases the patient hotel is a hotel where other patients and/or their relatives can stay near the hospital.). Negative remarks consisted of it being impersonal, isolated, and an inappropriate environment for the newborn baby. This model did not offer sufficient support for primiparas, and it was sometimes not even perceived as postpartum care at all.

*You feel lonely at a hotel. I mean, when you go to the dining room where there are cancer patients, relatives, staff etc, you've got to "be on you best behaviour", try to sit properly, walk properly, dress reasonably and so on. In the maternity ward everyone's in the same boat. You feel you have something in common with other women. You can sit on a ring without people staring at you. It's also nice to get to know new people*.

### Staff attitudes and behaviour

Avoiding making contact, not showing concern about the mother's feelings, and not asking about the baby's or the mother's health, was interpreted by the mothers as a *lack of interest *in them and their family. Staff who persistently asked them to leave, sometimes despite medical complications, or who expressed relief and happy feelings when the family was about to go home, made the mother feel unimportant and *invisible*.

*In the two days I was at the maternity ward I hardly saw any of the staff, and nobody asked how my daughter was*...

*They were happy to get rid of us at the maternity ward. "Good, that gives us an empty bed", was what they said when we left. It was bad to say that, we thought*.

Personnel were at times seen by women to be working merely according to routine. The midwives are *sometimes more interested in their coffee breaks than their patients, unfortunately*

*Nurses seem to just repeat well-practised phrases. Deeper questions remain unanswered*.

Descriptions of caregivers as *insensitive, unfriendly, disrespectful and impersonal *were reported. The perception that some staff were lacking in understanding and sympathy for exhaustion (tiredness) and breastfeeding difficulties undermined women's sense of being capable mothers. Caregivers were seen as insensitive and tactless when they acted in a grumpy, irritated manner, and hissed at the mother. Negative descriptions also related to situations when mothers felt that the staff were criticising them, or did not respect their own decisions. One woman complained when she felt that she was "treated like an idiot" on one occasion, and then expected to know everything.

They said: – Do you mean that you're so selfish that you can leave your child crying, because you need to go to the toilet?

In Sweden the main responsibility for care during the postpartum period rests with the midwife. However, when expressing negative experiences with staff in Paper IV, women referred to several different professional groups, such as midwives, nurses, doctors, assistant nurses and physiotherapists.

Some women were concerned about the *incompetence *of the staff. Negative descriptions were related to lack of competence and knowledge about treatment and medical complications.

*... My midwife has been helpful, but nobody seems to know exactly what they should do, and how you can form an opinion. They asked the staff at the Gynaecological Clinic, but they had no idea. I mean, you'd think that the midwife should know how to get rid of it anyway*...

Negative comments also related to the time constraints of the staff, which made them *rushed, stressed *and sometimes even confused and mixed up. Some parents felt reluctant to ask questions because of the high stress level of the staff.

*... in most cases somebody looked in when they changed shifts, but not always. There was no time/interest for a chat. If I had an urgent, concrete question (preferably a medical one), I got an answer. But I would really have appreciated it if I'd been given time for those small questions that you bring up if you feel that there's time for them. They told me to come out and look for one of the staff, if I wanted something urgently. Even if I already had one child, you still need to go through some things again, and new questions come up. I think quite simply that it was the care itself that was lacking. At the same time you keep hearing about the crisis in health care, so it feels difficult to make demands over and above the medical questions*.

### Breastfeeding support

Breastfeeding support was a major issue of concern. Negative statements were linked to experiences of insufficient support and lack of helpful advice. *Insufficient support *concerned general matters as well as specific areas, e.g. breastfeeding after a breast operation, a caesarean section or in cases when the baby was underweight. Being a "baby-friendly clinic" and at the same time forcing mother and baby to go home early was seen as contradictory. Problems with insufficient milk supply and supplementary feeding were seen as forgotten subjects on the postpartum wards of today. Incorrect instructions and guidance led to anger, worry and disappointment. Inaccurate support comprised monotonous, simplistic and poor standard of breastfeeding knowledge.

*What I'd like to see is more information and knowledge on the part of the midwife, about breastfeeding for women who've had breast operations. Nobody can give tips or advice about what to do to get the breastfeeding going in the best way. I've had to read about it in newspapers afterwards, when it was too late and the baby had already got into the habit of preferring the bottle for most meals. You get into a vicious circle when you don't know what to do, and it has a bad effect on your relationship. Most people just say "that's the way it usually is" and "you'll probably have to use supplements", but nobody actually knows anything*.

*Inappropriate support *included too much and wrong focus on breastfeeding supervision, lack of respect and understanding of the mother's own decision, and exaggerated concentration on the advantages of breastfeeding. Part of this sensitive issue evolved around feelings that pressure, guilt and blame was put on mothers who were using supplements. Mothers expressed that they wanted to be encouraged instead of pressured.

*... Something that was difficult at the maternity ward was that they went on and on about breastfeeding. You were supposed to ring for the midwife when you breastfed so as to "pass the test", If the baby stopped to catch its breath for a moment when the midwife came in, she started to poke my breast and show what to do, but the baby had been sucking the second before, so I knew that the baby got fed. Some of the midwives' comments made you feel like a bad mother, although deep down you knew that the breastfeeding was going OK*.

*I think there's too much pressure on new mothers when it comes to breastfeeding. Everybody goes on about how important breastmilk is, and of course it is important, but the fact that "everyone" goes on about it means that you easily get performance anxiety. It takes a while before it works properly, but all the nagging makes you feel that you have to make it work right away. I think everyone knows that breastmilk is the best thing for the baby. But if it doesn't work there are other alternatives. The staff at the maternity ward should encourage the new mother to feel good. Also to use supplements if that's the way the mother wants it, and not to be so judgemental if she wants to combine breastfeeding and breast milk substitutes*.

*They should also take breastfeeding a bit easier at the maternity ward and not frighten the mothers so much, just because it doesn't work right away. All this fuss about getting us to breastfeed made me really stressed*.

*I breastfed for ten weeks but it was difficult. I've never had very much milk for any of the children, all six of them. Sometimes I feel that I'm a bad mother because I don't breastfeed. There's too much talk about how positive it is for the baby and the mother. Anyone who can't breastfeed for some reason gets into a real state of anxiety. I certainly do anyway*.

### Information

*Insufficient information *was identified within the following areas: physical changes and adaptation, postpartum pain and pain relief, self-care (e.g. diet, exercise, pelvic floor exercises), emotional issues and psychological adjustments, adaptation to parenthood, the birth experience, and chid care (e.g. what a child needs, nappy changes, red bottoms, colic and baby crying). Insufficient information affected women both during the hospital stay and after discharge from hospital.

*I could have done with more information after the birth about for example how the body would react, what might happen etc. Everything has been a bit unreal up to the birth. Then our baby arrived, and all of a sudden that made everything else unnecessary, uninteresting and unimportant. Then I wanted to know everything about how I could best satisfy my baby*.

Some women said that there was a lack of standardised basic information.

*You've got to ask a lot yourself to get the information you need*.

During my stay at the maternity ward (which was very short) I felt that I got help and information if I asked, but I would have liked some sort of "basic information". That it wasn't just me who had to take the initiative to ask

*Inconsistent information *meant a setback for some women:

*Information at the maternity ward – depends a lot on the midwives – double messages. Some give information that isn't asked for or required*.

*You get a lot of different messages at the maternity ward. All of them have different opinions and as a new parent you only need to hear one answer. One midwife said one thing, the second didn't think you should do that and the third had yet another opinion. Perhaps they should discuss it a bit between themselves, so that they say more or less the same thing*.

*Incorrect information *sometimes led to complications or postponing of recovery. It also made the first period with the baby less enjoyable. Unrealistic information counteracted optimal adjustment.

*... I've had some problems with the stitches healing, 4 of them. Apparently I've got some sort of tissue that's grown in between the sutures... It's very painful and I go for treatment several days a week.... It just gets more and more painful after each treatment because they get closer to healthy tissue. What I mean is that I didn't get any information about what the stitches would feel like and how long it would hurt, so I put up with it for longer than I need to have done. Good painkillers or information about what it actually is would be a good thing*.

There's absolutely no information about how the nipples and the breast work.... Why should you have to suffer for such a long time simply because the information is terrible?

*If you're in the maternity ward I think someone ought to tell you that there are, for example, nipple shields. That was a tip that I got from a friend, and it saved me from total depression*.

### The role of the father

Women expressed disappointment that postpartum care was organised mainly around mother and baby, leaving the father outside *lacking attention and support*. Their partners rarely had the opportunity to talk to any caregiver themselves. Positive encouragement and information about the transition to parenthood for the fathers was scarce. Mothers described that the fathers felt forgotten and abandoned if they were not given the opportunity to get involved in postpartum care. Sending fathers home made them feel superfluous and unnecessary, and the mothers felt lonely and without practical help. The message about the importance of the father's involvement was seen by some as "lip service" only:

*In their information file at the maternity ward they said that it was important for the father to be there at the maternity ward and get to know his child, but they still sent him home*.

### Attention to the mother

Women expressed that *insufficient attention was paid both to their own physical health and emotional needs *during the postpartum stay in hospital. After the baby was born, they felt neglected because all focus shifted to the baby.

*... that the woman disappears from the picture after the birth (as far as certain information is concerned, and also health check-ups) after having had a central position during the whole pregnancy*.

Neglected areas were: management of medical complications, postpartum pain, breast complications, and emotional adaptation.

*I felt that the follow-up of my health at the maternity ward was forgotten. This led to long-term bleeding which became acute in the end*.

*I think it's beneath all criticism that they don't follow up a torn vagina. I'd like to have more help with how I look and feel*.

Why doesn't anyone tell you about afterpains! There must be better painkillers than Panodil for that! After about two hours I got such strong afterpains that I couldn't have my son on my lap! I felt so upset and disappointed because I thought that everything would have been over by then, and I should have been able to enjoy being with my little miracle and not be in such incredible pain!

Problems of not being taken seriously were mentioned, in relation to worries about both physical and psychological health. The use of what one woman called "the help-yourself model" left a sense of being forgotten and abandoned. Lack of support included insufficient contact with staff, lack of answers to questions asked, feeling "in the way", and a general lack of individualised care. If nobody asked if everything was okay, the women felt neglected.

*Nobody asked how I was. Just said hello. Told me about times for breakfast and so on. Otherwise they never came and checked how things were or asked if the breastfeeding was going OK. I think it was rather awful really*.

One woman wished she had "dared" to ask for help but did not because she wanted to prove that she was capable, and she did not want to bother the staff unnecessarily.

*... sort of want to show how capable they are and therefore don't ask for help too often at the maternity ward. You don't want to trouble them unnecessarily. Then when you've finally come home, it hurts and it's difficult to get help*.

Lack of attention to the mother, captures a broad range of experiences, including aspects of care partly described in the former categories. This overlap is illustrated in the following quotation:

*I think it was a bit "boring" at the maternity ward, you mostly feel in the way. I mean ideally EVERYONE is supposed to go home a few hours after the birth. Can't they show some consideration for how much help you have at home? Perhaps you need an extra day or so to feel that you can manage. I got inflammation of the balance nerve, so I mean I didn't really feel all that well. And it's because of the inflammation (I think), that the breastfeeding hasn't worked so well to start with. I was just too tired and "weird" in the head. Felt like a bad mother who had to give my baby supplements*.

### Positive comments

Since exploration of positive experiences are informative and may help improve care as well, these are briefly presented. Often women did not specify why they were satisfied. The descriptions were broad and general, but sometimes related to specific issues, such as care on the mother's terms, good supportive care, encouragement and help. Some women praised the staff as being extraordinarily competent, supportive, helpful and tactful. In other cases, women were grateful because they were discharged from hospital when they had wished to; or were related to having a room of their own where the baby's father could stay overnight. Privacy and independence at a postpartum hotel, the small scale of a postpartum ward, and satisfaction with pain relief, were also mentioned. The positive comments did not address any distinct area that was not also mentioned in the negative statements.

## Discussion

The focus of this study was on areas of non-optimal care, based on experiences that could help in improving hospital care and follow-up after childbirth. Therefore, we do not give a comprehensive picture of Swedish postpartum care, since only negative statements are analysed. The rationale for giving information about how often a category was mentioned by the respondents was to give an idea of the significance of this category. However, such numbers do not necessarily illustrate the magnitude of a certain problem in this context of qualitative data.

The study group was more often cared for in a large hospital (> 4000 deliveries/year), and they had less opportunity to talk through their birth experience, compared with women who did not give any negative statements. The finding that 51 % rated postpartum care overall as either "very positive" or "positive" suggests that a critical comment on a certain aspect of care does not exclude a general positive assessment. The spontaneous comments about postpartum care were in general rather severe, and the fact that so many women made a positive overall assessment in spite of these comments, strengthens the view that patients are reluctant to be critical about the care they receive, when asked for overall single ratings care [9-12].

In order to better understand the findings of this study we will discuss them in relation to changes that have taken place in Swedish postpartum care over recent decades. In 1960, the aims of postpartum care were defined by the Swedish National Board of Health and Welfare as being: 1) initiation of breastfeeding; 2) maternal rest; 3) learning to care for the baby; and 4) increased infant weight [39]. The recommended number of days in hospital after a normal birth was 7 to 8. At that time, postpartum care was characterised by strict and rigid routines, such as scheduled breastfeeding every fourth hour during daytime, and supplements or other mothers' breast milk in the nursery at night. The mother's uterus and bleeding was checked regularly, visiting hours were restricted, and the baby's father was treated as any other visitor. Since then, dramatic changes have taken place. The nurseries have been closed down, and mother and baby room-in day and night. Scheduled breastfeeding has been replaced by breastfeeding on demand, visiting hours are more flexible, and fathers can stay at the postpartum ward during daytime, and in many places around the clock during the entire stay. The number of women in each room has been reduced, from 4–6 in some hospitals to 1 or 2. Another dramatic change is the reduction in the number of beds in the postpartum wards, and the ensuing reduction in the length of stay. In 2004, the average length of stay after a vaginal delivery was 2.28 days [40]. All these changes in the Swedish system have been rather similar to those taking place in other countries of the same socioeconomic level. One difference is that postpartum follow-up at home has not been part of standard care in Sweden [41], as, for instance, in the UK and Australia.

The findings of our previous quantitative study of women's overall assessment of postpartum care showed that 26% of the women were not satisfied, a much higher figure than the one related to antenatal or intrapartum care [4]. Unfortunately we have no reason to believe that postnatal care has improved since the data collection of the KUB study. On the contrary, a recent Swedish study suggests that dissatisfaction with postnatal care has increased. Maternal overall dissatisfaction with postnatal care was 34% in a more recent Swedish study [42].

The present study, based on the women's spontaneous comments, adds to this information a wide range of specific issues which make women uncomfortable with their postpartum experience. These findings raise important questions, such as: Were women more satisfied with postpartum care some decades ago? How can today's postpartum hospital care and follow-up be improved?

We did not find any Swedish study describing women's experiences of postpartum care some decades ago. However, an interview study where women in different age groups were asked to rate retrospectively their experiences of care during childbirth suggests that satisfaction with maternity care in general has increased dramatically [41,43]. We believe that one explanation for these findings may be that women are treated more respectfully today than, for instance, fifty years ago. Nevertheless, the present study shows that the way women are treated is still an important issue. Threats to personal identity, i.e. a perception of being dehumanised, objectified, stereotyped, disempowered and devalued, seem to underpin almost all reports of negative experiences of health care, as suggested by Coyle [24]. The reason why this is a problem may, however, have changed. Whereas caregivers some decades ago were probably more authoritarian due to more hierarchical structures in hospitals and in society in general, caregivers today may treat patients inappropriately because they are under stress. Associations have, for example, been reported between the work environment at hospitals (e.g. adequate staffing levels, good administrative support and relations with doctors) and job burnout for nurses, and patient outcomes such as mortality and satisfaction [44-46].

Patient's frustration with issues such as shortage of staff, lack of accommodation or non-optimal timing of discharge may all be related to problems concerned with access of care [24]. Similarly, from the staff perspective, the short duration of the hospital stay and the large turnover of patients have made it more difficult to give the necessary information and support. This situation may also make it harder for caregivers to be sensitive to each woman's needs, and to respect women's individuality, personal knowledge and experience [24]. Women in the present study described a sense of dehumanisation regarding rule-breaking; that is, when staff broke unspoken "taken-for-granted" rules (e.g. listening carefully, not being aggressive, not interrupting, shouting or breaking in on privacy) [47]. When trying to understand why such rules are broken within medical settings it has been suggested that it relates to the way "work is organised", that staff usually do not know patients and that medical aspects of work are prioritised over psychological ones [47].

The inflexibility associated with the time of hospital discharge, was a problem for some women. They were dissatisfied when they were sent home involuntarily and when the ward was overcrowded. As stated earlier, the short duration of the hospital stay could also be an indirect reason why the staff did not have sufficient time to spend with each woman, to give necessary information and support, and to be available for the woman's own questions. The decreasing length of postpartum stay has been widely discussed internationally, and many attempts are being made to find alternative ways of supporting women, such as domiciliary visits, telephone follow-up, and clinic visits. Domiciliary visits may be one of the best ways of giving individualised care [48-50]. Two studies from California [49,50] showed that, for low-risk mothers and newborns, home visits compared with hospital-based follow-up were associated with equivalent clinical outcomes and higher maternal satisfaction, although home visits were more costly.

Even if domiciliary support of some kind may optimise the possibility of adapting care to the individual woman and her family, there could be improvements within the hospital environment. Problems with the hospital environment similar to those identified in our study were reported also in an American study [51], in which it was found that bothering stimuli, such as light and noise, lack of privacy and staff entering rooms unannounced, were particularly criticised issues. However, the impact of these negative factors could be modified if staff provided information about procedures and adjusted inconvenient conditions. In addition, the woman's own condition, e.g. postpartum complications, had an effect on how she managed to cope with the environment in the postpartum ward [51].

Whereas postpartum care in the old days was driven by rigid routines and medical check-ups, today's care may have moved too far in the opposite direction. Our findings suggest that some women feel that they are forgotten, and that all initiatives are handed over to them. Staniszewska and Henderson [9-12] explained this lack of attention in terms of the phenomenon "sense of engagement with the health care system" (p. 534). Disengagement occurred when patients did not feel that their needs, experiences and concerns were understood. Some women in our study even felt that their physical health and recovery after the birth was neglected. A challenge for the caregiver is to get the right balance between the amount of information given, medical check-ups and time for the mother herself to ask questions [52]. There is some evidence that the use of routine checklists, where the caregiver ticks all items done, does not meet the needs of the individual woman, but rather those of the caregiver [15].

Another problem raised by women in this study was feelings of guilt when breastfeeding was problematic. During the establishment of breastfeeding, different aspects of support such as inappropriate advice and lack of knowledge were reported as problems by mothers in this study. Supporting breastfeeding is one of the major tasks for midwives working on postpartum wards in Sweden, and this focus has also been very successful in terms of high rates of breastfeeding duration. Six months after birth, 72% of Swedish mothers still breastfeed [53]. However, it is also a challenge to balance breastfeeding information and support with a tolerant and respectful attitude to mothers who experience difficulties. Mothers' high sensitivity to critical comments and opinions of others regarding breastfeeding has been explained by the high emotional distress caused by breastfeeding difficulties [54], and by the fact that this triggers questions as to whether they are capable as mothers [55].

Another problem raised by women in this study concerned experiences related to the baby being in the neonatal unit. This was associated with different problems, such as increased stress, and uncertainty of what was expected of them during such a separation. This seems to be a problem which could be addressed relatively easily and immediately by better collaboration between the postpartum units and the neonatal clinics.

Yet another issue raised in this study was the role of the father. Fathers' involvement during pregnancy, childbirth and early parenthood has increased dramatically in Sweden. In 2006 70–75 % of the fathers used their right to take paternal leave during the first 10 days after the baby was born. This very positive development towards more committed fathers also affects assessments of postpartum care. Swedish fathers and their partners, request more and more that postpartum care should not only be for mother and baby, but for the entire family. Postpartum wards that allow fathers to stay overnight are more popular than those that do not [4]. However, staying at a hotel near the hospital, as an alternative mode of postpartum care, has not been as successful, even if one of the goals was to allow the family to stay together. The reasons given by women in this study were related to it being impersonal, isolated, and an inappropriate environment for the newborn baby.

Today's expectant and new parents differ from those of some decades ago, not only by being more equal, but also by being better informed, more aware of their rights and more used to expressing their views. This may impact on their assessments of maternity care, and increase their demands on the quality of care. In this context it is important that the aims of postpartum care are well defined. This is not the case in Sweden today. Apart from the initiation of breastfeeding, the written aims from 1960 are no longer relevant. Postpartum wards are not intended for maternal rest, and the infant's weight curve does not need to go up before discharge. The mother may get some teaching in how to care for the baby, but she is also expected to gain these skills on her own, or from others. Routine checking of the uterus is no longer regarded as necessary in a normal birth. Maybe clearer statements about what to expect from the hospital stay after the birth would lead to more realistic expectations and greater satisfaction with postpartum care.

### Method discussion

In this study we mainly performed manifest content analysis, i.e. concerning the obvious components of the text. Data quality did not allow for extensive latent analysis, i.e. a coding of the 'underlying' meaning and implied feelings [36]. Lack of persistence and personal preconceptions are potential threats of errors in the coding process; therefore the authors checked and discussed coding accuracy throughout the analysis.

Strengths of the study involved information about the participants' varied background characteristics, labour outcomes and experiences, which enhanced the possibility of shedding light on a range of critical postnatal experiences. Also, the units of analysis provided insight into experiences of postnatal care, without the researchers exerting direct influence on the informants, or 'interviewer bias' [56]. A disadvantage was that it was not possible to ask follow-up questions in order to clarify ambiguities. Another limitation was the lack of information about the views of those who chose not to answer the open-ended questions. Also, a greater variability in responses has been shown when postal questionnaires rather than interview methods are used; that is, respondents tend to express strong views, in one or the other direction [29]. On the other hand, postal questionnaires are associated with a minimum of pressure for 'socially acceptable' responses [29]. In our study, it may have been easier for some disadvantaged groups to reply, e.g. young, shy, distressed or early discharged women; and harder for others, e.g. women with writing or language disabilities. The quality of data varied from short, condensed and straight forward descriptions of dissatisfaction; to rich, detailed and comprehensive descriptions.

Balancing mutually exclusive categories and the right level of abstraction was sometimes problematic. Certain comments described a chain of events that affected each other. Access to care, for example, is a prerequisite for feeling helped and supported. Also, different aspects of how care was organised might explain why mothers felt neglected.

Data was collected at two different time points, two months and one year after the birth. The majority (n = 140, 93%) wrote their comments in the first questionnaire when memories and involvement in issues related to postnatal care were probably stronger than ten months later. Our data did not allow for any comparison between comments from the two time points, and we could therefore not explore whether women had become more critical over time, as has been suggested by some studies of women's experience of childbirth [57,58].

Transferability of results is limited because of the self-selected sample in this study. However, the context of the sample was well defined, by representing a sub-sample of the larger national "KUB" study (extensively described elsewhere [4,31-34]). Apart from a larger proportion of Swedish speakers and a lower rate of smokers, women in the KUB study were similar to all women who gave birth in Sweden in 1999. The present sample undoubtedly represents no more than a minority of the KUB women, but nevertheless their comments have identified problematic areas of postnatal care that may also be relevant to a larger population.

## Conclusion

The findings of this study underline the need to further discuss and specify the aims of postpartum care. The challenge of providing high-quality follow-up after childbirth is discussed in the light of a development characterised by a continuous reduction in the length of hospital stay, in combination with increasing public demands for information and individualised care. An awareness of mothers' specific needs is extremely important in order to modify information and breastfeeding support, and also to make appropriate clinical decisions and succeed with symptom management.

### Future studies

Future research is suggested to explore for whom and in what situations care requires improvement, and to develop strategies for increasing individual satisfaction in new mothers as well as fathers. Future studies on how to give information at postnatal wards, with a focus on information being evidence-based, appropriate and tailored towards individual needs, are also needed. Findings from this study reinforce the need for further studies and evaluation of care when staying at a hotel near the hospital, as an alternative mode of postpartum care.

## Competing interests

The author(s) declare that they have no competing interests.

## Authors' contributions

AR had the responsibility for study conception, data management, performed data analysis and interpretation and drafting of the manuscript. UW had the overall project responsibility, performed data analysis and interpretation and helped to draft the manuscript. All authors read and approved final manuscript.

## Pre-publication history

The pre-publication history for this paper can be accessed here:


